# Supplementary material for: Formulation of Tioconazole and Melaleuca alternifolia Essential Oil Pickering Emulsions for Onychomycosis Topical Treatment
Source: Molecules. 2020 Nov 26;25(23):5544. doi: 10.3390/molecules25235544 (PMC7730074; doi:10.3390/molecules25235544)
Supplement: Supplementary file 1 [file molecules-25-05544-s001.pdf]

Formulation of Tioconazole and *Melaleuca alternifolia* Essential Oil Pickering Emulsions for Onychomycosis Topical Treatment

Barbara Vörös-Horváth[1], Sourav Das[2,3], Ala′ Salem[1], Sándor Nagy[1], Andrea Böszörményi[4], Tamás Kőszegi[2,3], Szilárd Pál[1*] and Aleksandar Széchenyi[1*]

[1] Institute of Pharmaceutical Technology and Biopharmacy, Faculty of Pharmacy, University of Pécs, H-7624 Pécs, Hungary; barbara.horvath@aok.pte.hu (B.V.-H.), ala.salem@aok.pte.hu (A.S.), sandor.nagy@aok.pte.hu (S.N.)
[2] Department of Laboratory Medicine, Medical School, University of Pécs, H-7624 Pécs, Hungary; pharma.souravdas@gmail.com (S.D.); koszegi.tamas@pte.hu (T.K.)
[3] János Szentágothai Research Center, University of Pécs, H-7624 Pécs, Hungary
[4] Department of Pharmacognosy, Faculty of Pharmacy Semmelweis University, H-1085 Budapest, Hungary; aboszormenyi@gmail.com
* Correspondence with equal contribution: szilard.pal@aok.pte.hu (S.P.); szechenyi.aleksandar@gytk.pte.hu (A.S.); Tel.: +36-72-501-500 (23554) (S.P.); Tel.: +36-72-501-500 (28801) (A.S.)

For the synthesis of hydrophilic silica nanoparticles (HS) the mixture of ultrapure water, ethanol and tetraethoxysilane was stirred for 15 min. Then the reaction mixtures were sonicated for 15 min (Bandelin Sonorex RK 52H, BANDELIN electronic GmbH & Co. KG, Berlin, Germany), followed by the addition of 10 w/w% ammonium hydroxide solution under stirring. The reaction mixtures were stirred for 24 at constant temperature. The ethyltriethoxysilane were dissolved in ethanol and then added to the hydrophilic silica nanoparticle suspension, and the mixture was stirred for 6 h at room temperature (25°C). The ammonium hydroxide and ethanol content were always removed by fractional distillation (Laborota 4000; Heidolph, Schwabach, Germany) from the surface modified silica suspension, prior to emulsion preparation. The water content was supplemented three times. The concentration of silica nanoparticle water-based suspension was finally adjusted to 1 mg/ml.

Supplementary Table 1. Parameters of hydrophilic and surface-modified silica nanoparticle synthesis.

**Hydrophilic Silica Nanoparticle Synthesis**				
**Sample**	**c_{TEOS} (mol/dm3)**	**c_{NH_3} (mol/dm3)**	**c_{H_2O} (mol/dm3)**	**t (°C)**
20HS		0.097		30
50HS	0.26	0.194	5	40
100HS		0.1940		25
**Surface Modification of Silica Nanoparticles**				
	**Sample**	**V_{ETES} (µl)**		**t (°C)**
	20ET	110		25
	50ET	40		25
	100ET	22		25

Supplementary Table 2. HSPs of tioconazole and the three main components of Melaleuca alternifolia essential oil.

**Component**	**δ_d**	**δ_p**	**δ_h**	**δ**
p-cymene	17.4	2.27	2.44	17.72
terpinene-4-ol	17.26	4.05	7.15	19.12
γ-terpinene	16.6	1.69	3.68	17.09
tioconazole	20.59	6.87	5.44	22.38

Supplementary Table 3. Calculated solubility parameters of tioconazole compared to the three main components of *Melaleuca alternifolia* essential oil.

Tioconazole VS	Distance*	$\Delta\delta$	$\Delta\delta_d$	$\Delta\delta_p$	$\Delta\delta_h$
p-cymene	8.4	4.7	3.19	4.6	3
terpinene-4-ol	7.4	3.3	3.33	2.82	1.71
γ-terpinene	9.7	5.3	3.99	5.18	1.76

*HSP Distance is a numerical value generated by the software that describes the distance between molecules in the Hansen space.

Supplementary Table 4. Calculated solubility parameters.

Solvent 1	Solvent 2	Solvent 3	Vol% 1	Vol% 2	Vol% 3
p-cymene	terpinene-4-ol	γ-terpinene	18	82	0
p-cymene	terpinene-4-ol	γ-terpinene	46.7	43.2	10.1
